# The effects of endoscopic-guided balloon dilations in esophageal and gastric strictures caused by corrosive injuries

**DOI:** 10.1186/1471-230X-13-99

**Published:** 2013-06-10

**Authors:** Yi-Chun Chiu, Chih-Ming Liang, William Tam, Keng-Liang Wu, Long-Sheng Lu, Ming-Luen Hu, Wei-Chen Tai, King-Wah Chiu, Seng-Kee Chuah

**Affiliations:** 1 Division of Hepato-Gastroenterology, Department of Internal Medicine, Kaohsiung Chang Gung Memorial Hospital and Chang Gung University College of Medicine, 123 Ta Pei Road, Niao Sung District, 833, Kaohsiung, Taiwan; 2 Lyell McEwin and Royal Adelaide Hospitals, Adelaide, Australia

**Keywords:** Esophageal stricture, Gastric outlet obstruction, Corrosives, Balloon dilation

## Abstract

**Background:**

Esophageal stricture (ES) and gastric outlet obstruction (GOO) can occurred in patients injured by the ingestion of corrosive agents. These complications may occur concurrently but has not been reported in the literature. The aims of this study are to assess the effects and complications of endoscopic-guided balloon dilations (EBD) in patients with corrosive-induced upper gastrointestinal strictures, either ES or GOO alone and simultaneous occurrences of both (ES + GOO).

**Methods:**

From July 2002 to December 2009, 36 patients with corrosive-induced upper gastrointestinal strictures in a tertiary hospital were recruited into this study. The patients were divided into three groups, ES group (n = 18), GOO (n = 7), and ES + GOO group (n = 11). All strictures were dilated under direct visualization by using through-the-scope balloon catheters to the end point of 15 mm. The end-point of treatment was successful ingestion of a solid or semisolid diet without additional dilation for more than 12 months.

**Results:**

These 36 patients included 15 males and 21 females with average age of 47 years ranging from 25 to 79 years. The success rates for ES group is significantly better than GOO and ES + GOO group (83.3% vs. 57.1% vs. 36.4% p = 0.035). Less complications were observed in ES group than in GOO and ES + GOO group (16.7% vs. 42.9% vs. 36.4%, p = 0.041). GOO group needed more sessions of dilations in order to achieve success dilations than ES and GOO groups (13.7 ± 4.9 vs. 6.1 ± 4.7 vs. 5.5 ± 2.1, p = 0.011).

**Conclusions:**

Corrosive injuries complicated with ES can be effectively and safely treated by EBD. However, the success rates declined significantly in patients with GOO with or without ES and amore complications occurred.

## Background

The ingestion of corrosive agents can cause extensive damage to the gastrointestinal tract. This can lead to significant morbidity requiring prolonged and repeated hospitalization. In the acute stage, the damage may be so severe that perforation of the esophagus and the stomach as well as death can ensue [1]. Long-term complications of the gastrointestinal strictures, including esophageal stricture (ES) and gastric outlet obstruction (GOO), may develop from weeks to years after ingestion of corrosive agents [2]. ES and GOO are considered different entities, but in patients injured by the ingestion of corrosive agents, they may occur independently or they may occur concurrently in up to 20% [3]. Endoscopy can be used to assess the degree and extent of damage of gastrointestinal tract within the first 48 hours, and later it can also be used to treat strictures developing in the esophagus and stomach [4-6]. Previous studies have reported the successful use of the endoscopic balloon dilation (EBD) to treat corrosives-induced ES or GOO in isolation [6-9]. In contrast, the use of EBD to treat patients who have both ES and GOO has not been formally evaluated. Once they occur concurrently, endoscopic treatment can be more complicated. The aims of this study are to assess the effects and complications of endoscopic-guided balloon dilations (EBD) in patients with corrosive-induced upper gastrointestinal strictures, either ES or GOO alone and simultaneous occurrences of both (ES + GOO).

## Methods

From July 2002 to December 2009, patients with ES and GOO caused by corrosive injury in a university-affiliated tertiary care center were recruited into this study. These patients had received early upper gastrointestinal endoscopy (GIF-Q240; Olympus Optical Co., Ltd., Tokyo, Japan) within 48 hours of ingestion. Mucosal burns of the esophagus, stomach, and duodenum were graded following a method previously reported by Zargar et al.: grade 0, normal examination; grade I, edema and hyperemia of the mucosa; grade II, subdivided into grade IIa (friability, hemorrhages, erosions, blisters, whitish membranes, exudates and superficial ulcerations), grade IIb (grade IIa plus deep discrete or circumferential ulceration), and grade III, multiple ulcerations and areas of necrosis [10]. If the patients demonstrated symptoms of upper gastrointestinal stricture, including dysphagia or easy satiety with postprandial vomiting, endoscopy was performed at four week after corrosive injury to examine the upper gastrointestinal tract. EBD was performed subsequently to patients with ES and GOO who satisfied the selection criteria by using through-the-scope balloon dilators. If active ulceration was noted within narrowing segment, the dilation procedure would be postponed and reevaluated two weeks later. Other exclusion criteria were (1) patients who had not perform an early endoscopy within 48 hours of ingestion, (2) patients who demonstrated symptoms of stricture but no upper gastrointestinal stricture on endoscopic examination, and (3) patients who decided to receive surgical intervention but not EBD. The patients were then divided into three groups esophageal stricture alone (ES), gastric outlet obstruction alone (GOO) and combination of ES and GOO (ES + GOO).

### Endoscopic balloon dilation

With informed consent of each patient, ES and GOO were dilated under direct visualization by using controlled radial expansion (CRE) balloon catheter (Microvasive, Boston Scientific Corporation, Natick, MA, USA) but without guide wire and fluoroscopic guidance. Intramuscular hyoscine butylbromide 20 mg as an antispasmodic agent and intramuscular meperidine hydrochloride 50 mg as an analgesic agent was given approximately 10 minutes before staring the procedure unless contraindicated. Before dilation, the diameter of stricture was estimated by comparing with an open biopsy forceps. Then we selected a balloon catheter according to the diameter of stricture and negotiated the balloon catheter through the working channel of the endoscope across the stricture without fluoroscopic monitoring. The balloon was inflated with water to the recommended pressure for 60 seconds. In each session, the patient received three consecutive dilations with increment of dilation diameter not more than 3 mm following the rule of three [11]. The stricture length of ES and GOO was measured from distal end to proximal end after dilation while withdrawing the endoscope. The patients were kept fasting for four hours after the procedure and proton pump inhibitors were prescribed to suppress gastric acid. Inpatients received two sessions a week and outpatients one session a week. Serial dilations were performed by gradually increasing the balloon diameters up to a maximum of 15 mm until solid or semisolid food could be tolerated. If GOO were encountered after ES was dilated, subsequent EBD for GOO was performed. Under such circumstances, the dilations of ES and GOO were counted together in one session. If symptoms of stricture recurred, additional dilations were performed until symptoms were relieved again. The treatment outcome was considered successful when patients were able to maintain a solid or semisolid diet without having to perform an additional dilation for the next 12 months.

### Clinical follow-up

Patients were treated with antacids or proton pump inhibitors for gastric acid suppression after each dilation session. Symptoms such as dysphagia and postprandial fullness sensation were recorded for each patient during the follow-up periods. Repeat dilations were performed for those patients with symptom relapse and proven to be stricture recurrences on clinical follow-up.

### Study definitions

The treatment success was reached when patients could ingest solid or semisolid diet for more than 12 months without additional dilation needed. The presence of any untoward event after endoscopic treatment was considered a complication such as gastrointestinal tract perforation or bleeding with clinical signs of hematemesis, coffee-ground vomitus, hematochezia, or melena, or significant pain requiring hospitalization, was defined as major complications. This study was approved by both the Institutional Review Board and Ethics Committee of Chang Gung Memorial Hospital (98-2106B).

### Statistical analysis

Continuous variables are given by mean and standard deviation. The continuous variables were analyzed by using the Mann–Whitney *U* test. Categorical variables were given in total and as percentages. They were analyzed by using the Fisher’s exact test. Two-sided *P* value of < 0.05 was considered significant. All statistical operations were performed using SPSS WIN version 15.0 (SPSS Inc., Chicago, IL, USA).

## Results

A total of 43 patients developed intake problems after ingestion of corrosives. Thirty-six patients were recruited into this study after excluding patients who received surgical management (n = 4) and those without stricture on endoscopic examination (n = 3). Among these 36 patients, there were 15 males and 21 females with average age of 47 years ranging from 25 to 79 years. The patients were divided into three groups, ES (n = 18), GOO (n = 7) and ES + GOO (n = 11) (Table 1). All strictures were dilated under direct visualization by using through-the-scope balloon catheters to the end point of 15 mm. There was no significant difference in age, gender and ingested substance (acid/alkali) among these three groups. Grade III injury over stomach was more common in those patients with GOO including GOO and ES + GOO group than those with ES alone (18/18, 100% vs. 8/18, 44.4%, *P* = 0.001).

**Table 1:** Clinical parameters and early endoscopic findings of patients with varied corrosive gastrointestinal strictures

	**ES**	**GOO**	**ES + GOO**	***P *value**
	**(n = 18)**	**(n = 7)**	**(n = 11)**	
Age, mean + SD (years)	44.5 ± 16.3	52 ± 16.9	47.3 ± 12.2	NS*
Male/Female	7/11	3/4	5/6	NS*
Acid/Alkali	15/3	7/0	11/0	NS*
Percentage of grade III injury				
Esophagus (%)	7 (38.9)	3 (42.8)	6 (75)	NS*
Stomach (%)	8 (44.4)	7 (100)	11 (100)	0.001
Duodenum (%)†	3 (16.7)	2 (33.3)	1 (20)	NS*

*no significance among varied groups.

†operators refrained from forcing the scope through the pylorus in 7 cases because of severe gastric damage.

Abbreviations: *NS* no significance; *ES* esophageal stricture; *GOO* gastric outlet obstruction; *ES + GOO* concurrent esophageal stricture and gastric outlet obstruction; *SD* standard deviation.

### ES group

Of the 18 patients with ES alone, 6 had orifices of strictures located in the upper third of the esophagus, 6 in the middle third, and 6 in the lower third. The mean length of stricture was 4.1 ± 1.5 cm (range 2 cm to 7 cm). Fifteen patients (15/18, 83.3%) had persistent symptom relief (average follow-up 25.5 ± 10.6 months). These patients received a total of 92 sessions of dilations with an average of 6.1 ± 4.7 sessions per patient over a median period of follow-up duration of 10 ± 15.9 weeks. Treatment failure was encountered in 3 patients (16.7%). One suffered from esophageal perforation after EBD and two opted out of dilation owing to refractory symptoms even after serial dilations (8 and 11sessions). All of them underwent surgical treatment with success.

### GOO group

Seven patients with GOO were found to have strictures located in the gastric antrum. The mean length of stricture was 2.5 ± 1.0 cm (range 1 cm to 4 cm). Four patients (4/7, 57.1%) were successfully dilated with persistent symptom relief. The average follow-up duration was 30 ± 15.8 months. These patients received a total of 22 dilation sessions with an average of 5.5 ± 2.1 sessions per patient over a median follow-up period of 6.0 ± 1.0 weeks. The other three patients suffered from EBD-induced perforations over channel of GOO (3/7, 42.9%). They were all treated with subtotal gastrectomy successfully without further surgical complication and were safe and sound.

### ES + GOO group

Of the eleven patients with ES + GOO, three had orifices of ES located at upper third section of the esophagus, four at the middle third, and four at the lower third. The orifices of GOO were all located over the antrum. The mean length of stricture was 3.6 ± 1.1 cm (range 2 cm to 6 cm) for ES and 2.4 ± 0.8 cm (range 1 cm to 4 cm) for GOO. Four patients (4/11, 36.4%) achieved treatment success with sustained symptom relief over an average follow-up period of 35 ± 27.2 months. These 4 patients received a total of 55 dilation sessions with an average 13.8 ± 4.9 sessions per patient over a median follow-up period of 21.0 ± 15.1 weeks. Seven of them failed after ERB (7/11, 63.6%). Major complications occurred after EBD in four of them (4/11, 36.4%). Two of them suffered from perforations over channel of GOO and two with active bleeding. All of them received subtotal gastrectomy. The other 3 patients (3/11, 27.3%) opted out of dilations owing to refractory symptoms even after serial dilations (10, 12, 13 sessions). All of them underwent surgical treatment either received gastrojejunostomy and esophagectomy with colon interposition without further surgical complications.

As shown in Table 2, the success rates for ES group is significantly better than GOO and ES + GOO group (83.3% vs. 57.1% vs. 36.4% p = 0.035). Less complications were observed in ES group than in GOO and ES + GOO group (16.7% vs. 42.9% vs. 36.4%, p = 0.041). GOO group needed more sessions of dilations in order to achieve success dilations than ES and GOO groups (13.7 ± 4.9 vs. 6.1 ± 4.7 vs. 5.5 ± 2.1, p = 0.011).

**Table 2:** Comparisons of the outcomes of endoscopic balloon dilation in patients with varied corrosive gastrointestinal strictures

	**ES**	**GOO**	**ES + GOO**	***P *value**
	**(n = 18)**	**(n = 7)**	**(n = 11)**	
Achieving persistent symptom relief, No.	15 (83.3%)	4 (57.1%)	4 (36.4%)	0.035
Major complication induced by EBD, No.	1 (5.6%)	3 (42.9%)	4 (36.4%)	0.041
Sessions of dilation to achieve persistent symptom relief, mean + SD*	6.1 ± 4.7	5.5 ± 2.1	13.7 ± 4.9	0.011

* For patients with effective outcome.

Abbreviations: *ES* esophageal stricture; *GOO* gastric outlet obstruction; *ES + GOO* concurrent esophageal stricture and gastric outlet obstruction; *SD* standard deviation.

The reasons of treatment failure were perforations (n = 6, 16.7%), ineffective dilations (n = 5, 13.9%) and active bleeding (n = 2, 5.6%). The overall incidence of major complications was 3.3% per dilation session (8/239), including 2.5% (6/239) with perforation and 0.8% (2/239) with bleeding.

## Discussion

Endoscopy should be avoided within 2 weeks after endoscopic balloon dilation because of the high risk of perforation [10]. However, there is no good evidence in the literature to suggest the best timing to perform endoscopic balloon dilation. EBD can be performed effectively and safely from four to six weeks after corrosive injury and is the treatment of choice for most of these injuries [5,11-13]. In patients with ES, esophagectomy followed by reconstruction operation can be performed, but such invasive procedure is grueling for both the patients and their surgeons. They should only be considered in severe complications, when EBD fails or when patients are unable to tolerate EBD procedures. Unlike ES, surgical intervention for GOO which usually involves subtotal gastrectomy or bypass gastrojejunostomy, is not so arduous and can be performed with relatively few complications 0% to 10.7% [14,15]. Therefore, surgery had been used as a standard treatment of caustic-induced GOO [14-16]. Recently, Kochhar and colleagues suggested that EBD was also a safe and effective treatment option in patients with corrosive-induced GOO and reported that persistent symptom relief could be successfully achieved in 95.1% of their patients with a extremely low perforation rate (2.4%) [6]. However, the overall success rate including both GOO group and ES + GOO group in our study was only 44.4% (8/18). One reason for this discrepancy might be that four patients (22.2%) could not tolerate this procedure and opted out of dilation. All of these four patients belonged to ES + GOO group. Since more dilation sessions were needed to treat patients with ES + GOO, these patients may have felt less inclined to continue the course of treatment. Another reason should be that patients with GOO in our study had more dilation-related major complication (7/18, 38.9%), which reduced their chances for successful outcomes.

**Figure 1:**
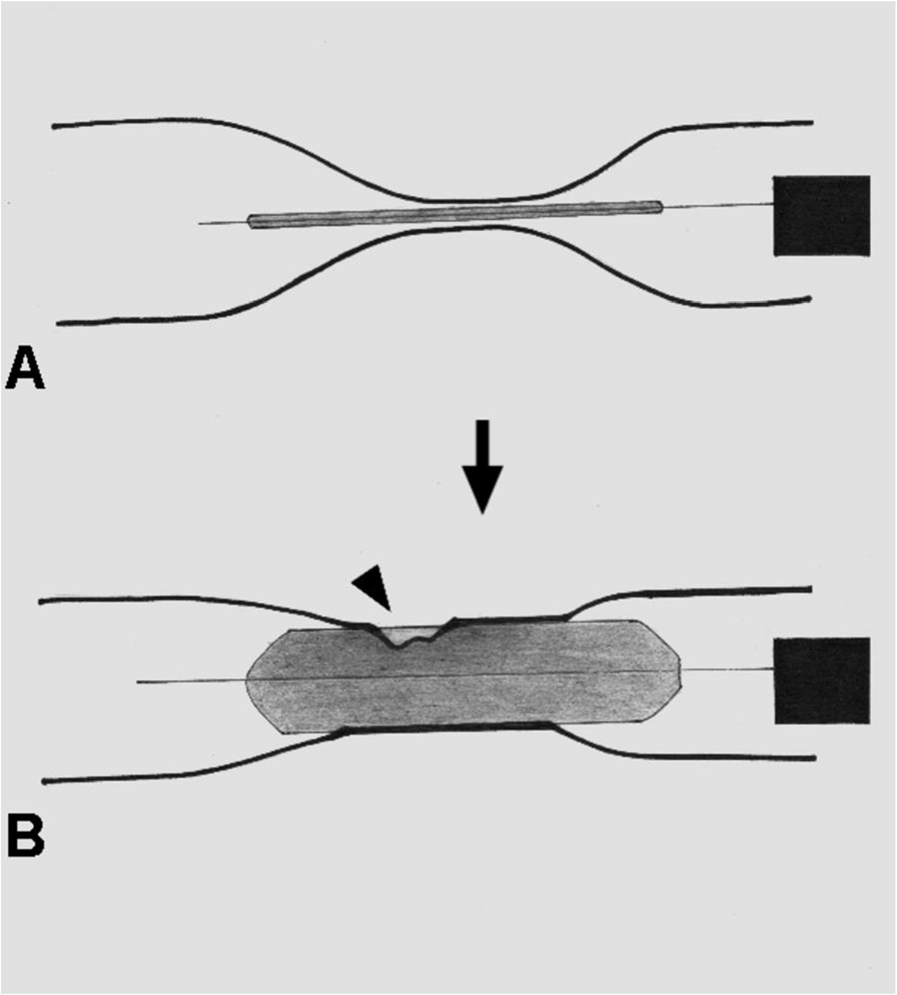
**The development of perforation in case of straight stricture (A) before and (B) after inflating balloon catheter.** The perforation (arrow head) may occur at the narrowest site.

**Figure 2:**
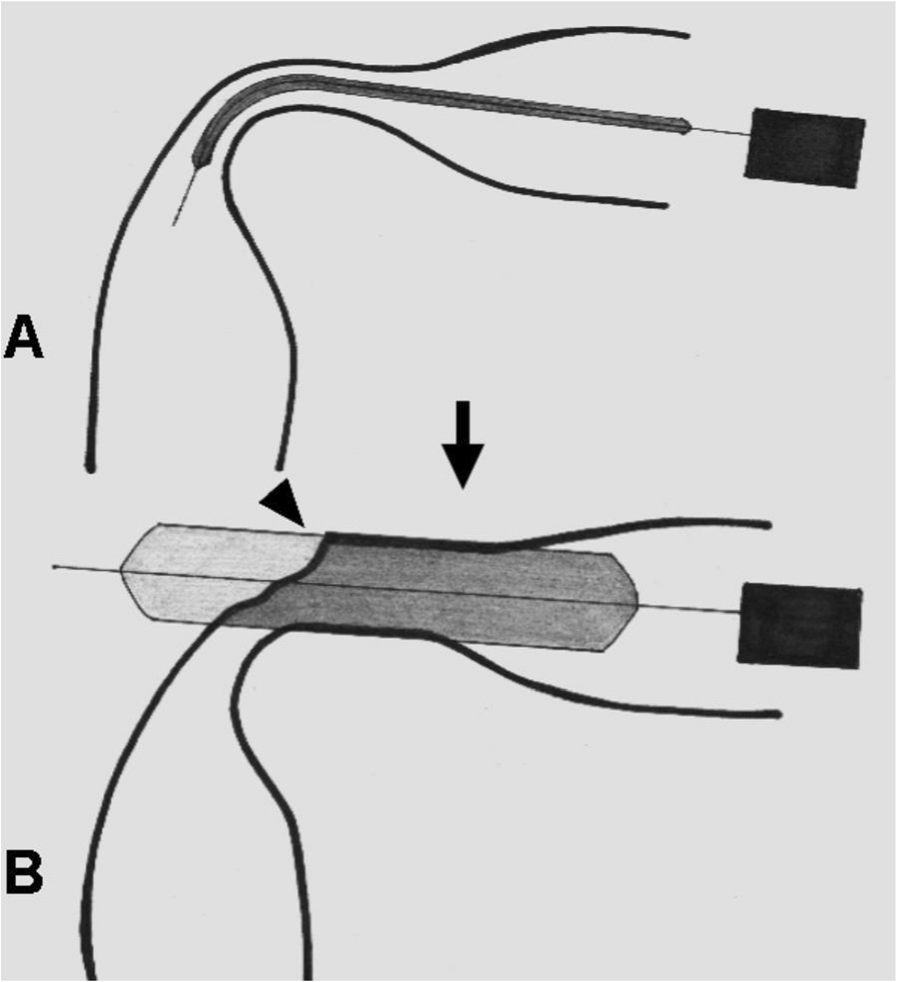
**The development of perforation in case of angulated stricture (A) before and (B) after inflating balloon catheter.** The perforation (arrow head) may occur at the angulated curve.

In our series, perforations were the major cause of treatment failure (6/13, 46.1%), including five in GOO and one in ES. These complications may occur when (1) inflating a balloon catheter within a straight stricture induces perforation caused by severe laceration on the narrowest area of the stricture during radial expansion (Figure 1) or (2) inflating a balloon catheter within an angulated stricture erects it forward and perforates the distal end of the angulations instead of curving at the corner (Figure 2). Lacerations of the narrowest areas of the stricture mainly occur when balloon diameters were overestimated. Experienced endoscopists chose the first balloon diameter based on the diameter of the stricture. Subsequent dilations are usually based on the “rule of 3,” referring to no greater than 3 consecutive dilators in increments of 1 mm per session [17]. If the balloon diameter is chosen this way, lacerations of this kind could be avoided. This could explain the perforation which occurred to our patient in the ES group. However, when an angulated stricture is encountered, a soft deflated balloon catheter can negotiate the angle. But once the balloon is being inflated, the bent end may rise up and tear the distal end of the stricture, especially if the stricture is at a sharp-angle (Figure 2). When the angle is sharp, even a careful approach still can damage the wall. The caustic GOO is often located in a curved area such as the pylorus or duodenal bulb. The corrosive agents may have deformed the antrum making the stricture even more angulated. The increased angulations could be the reason for more perforations in the GOO group than in the ES group in current study.

## Conclusion

In conclusion, Corrosive injuries complicated with ES can be effectively and safely treated by EBD. However, the success rates declined significantly in patients with GOO with or without ES and more complications occurred. Moreover, patients with concomitant ES and GOO require more EBD sessions than patients with ES or GOO alone to achieve long-lasting relief of their symptoms.

## Competing interests

The authors declare that they have no competing interests.

## Authors’ contributions

YCC (first author) carried out the analysis and interpretation of the data; drafting of the article. SKC, CML, LSL, MLH, WCT,and KWC participated in the critical revision of the article for important intellectual content. WT corrected the grammar deficiencies of this manuscript. KLW (corresponding author) conceived of the study and carried out the final approval of the article. All authors read and approved the final manuscript.

## Pre-publication history

The pre-publication history for this paper can be accessed here:

http://www.biomedcentral.com/1471-230X/13/99/prepub
